# One Shot, One Rhythm: Termination of Refractory Persistent Atrial Fibrillation in a Young Patient via Single Pulmonary Vein Application: A Case Report

**DOI:** 10.3390/jcm14207297

**Published:** 2025-10-16

**Authors:** Jonasz Kozielski, Alicja Dąbrowska-Kugacka, Ludmiła Daniłowicz-Szymanowicz, Marek Szołkiewicz

**Affiliations:** 1 Department of Cardiology and Interventional Angiology, Kashubian Center for Heart and Vascular Diseases, Pomeranian Hospitals, 84-200 Wejherowo, Poland; jonaszkozielski@gmail.com (J.K.); e.mars@wp.pl (M.S.); 2 Department of Cardiology and Electrotherapy, Medical University of Gdansk, 80-214 Gdansk, Poland; ludmila.danilowicz-szymanowicz@gumed.edu.pl

**Keywords:** persistent atrial fibrillation, pulmonary vein isolation, micro-reentry, tachyarrhythmia-induced cardiomyopathy (TIC)

## Abstract

**Background/Objectives:** Atrial fibrillation (AF) is the most common sustained arrhythmia, with catheter ablation outcomes differing significantly between paroxysmal and persistent forms. While pulmo-nary vein isolation (PVI) remains the cornerstone of ablation, persistent AF is often associ-ated with atrial remodeling and non-pulmonary vein triggers, reducing procedural success rates and necessitating repeat interventions. However, in selected patients with minimal atrial substrate, a single PVI may achieve durable rhythm control. This case report illus-trates such a scenario in a young patient with persistent AF and tachyarrhythmia-induced cardiomyopathy (TIC). **Methods:** A 42-year-old previously healthy male presented with newly diagnosed persistent AF complicated by TIC and heart fail-ure (left ventricular ejection fraction [LVEF] 25%). Despite rate control, anticoagulation, guideline-directed heart failure therapy, amiodarone pretreatment, and two failed electrical cardioversions, the patient remained symptomatic. Elec-troanatomic mapping was performed to assess atrial substrate prior to radiofrequency ablation. **Results:** Mapping revealed no extensive low-voltage zones, indicating absence of significant atrial fibrosis. During ablation, si-nus rhythm was restored spontaneously with a single application targeting the infero-posterior aspect of the right infe-rior pulmonary vein. No additional arrhythmogenic substrate was identified. The patient maintained sinus rhythm throughout 14 months of follow-up, with marked clinical improvement, normalization of LVEF (55%), regression of atrial and ventricular enlargement, and resolution of heart failure symptoms. Quality of life, assessed by the ASTA question-naire, improved from 24 to 0 points. **Conclusions:** This case highlights that even in therapy-resistant persistent AF with severe structural and functional cardiac impairment, arrhythmia may be driven by discrete pulmonary vein-dependent mechanisms. Careful patient selection, particu-larly in younger individuals without advanced atrial remodeling, can identify those in whom PVI alone achieves durable rhythm control and reverse cardiac remodeling.

## 1. Introduction

Atrial fibrillation (AF) is the most common sustained cardiac arrhythmia worldwide, with an estimated prevalence rising from 33.5 million in 2010 to approximately 59 million by 2019 [1]. Catheter ablation via pulmonary vein isolation (PVI) is an established treatment for AF, with significantly higher success rates in patients with paroxysmal AF compared to those with persistent forms. Long-term freedom from arrhythmia after a single procedure is reported in 70–80% of patients with paroxysmal AF, while outcomes in persistent AF are more modest, ranging from 50 to 65%, and often requiring repeat ablation [2,3]. These differences are attributed to structural remodeling, substrate complexity, and non-pulmonary vein triggers in persistent AF [2,3].

There is growing interest in the emerging concept of ‘PVI-dependent’ versus ‘PVI-independent’ AF, which, although not yet formalized in international guidelines, has increasingly been used in recent studies to stratify patients based on their response to ablation and the presence of atrial substrate beyond the pulmonary veins [4]. This assessment primarily relies on the identification of low-voltage areas or fibrotic zones in the left atrium, with data suggesting that approximately 90% of patients with paroxysmal AF are free of such substrate, compared to only 65% of those with persistent AF [5]. Patients with persistent AF who lack identifiable atrial substrate appear to respond more favorably to a single, empirical pulmonary vein isolation procedure [6].

Our clinical case demonstrates a patient with persistent AF and clinical symptoms of tachyarrhythmia-induced cardiomyopathy (TIC), refractory to permanent antiarrhythmic therapy (including amiodarone) and electrical cardioversion (ECV), in whom no clear extensive atrial substrate was identified on electroanatomic mapping, and sinus rhythm was restored during ostial isolation of the infero-posterior aspect of the right inferior pulmonary vein.

## 2. Materials and Methods

This is a report on a young patient with symptomatic HFrEF and co-existing permanent AF in whom (despite clinical poor characteristic) AF ablation in regard to pulmonary vein isolations turned out to be a highly effective procedure with long-lasting positive remodeling and outcomes.

## 3. Case Report

**Figure 1:**
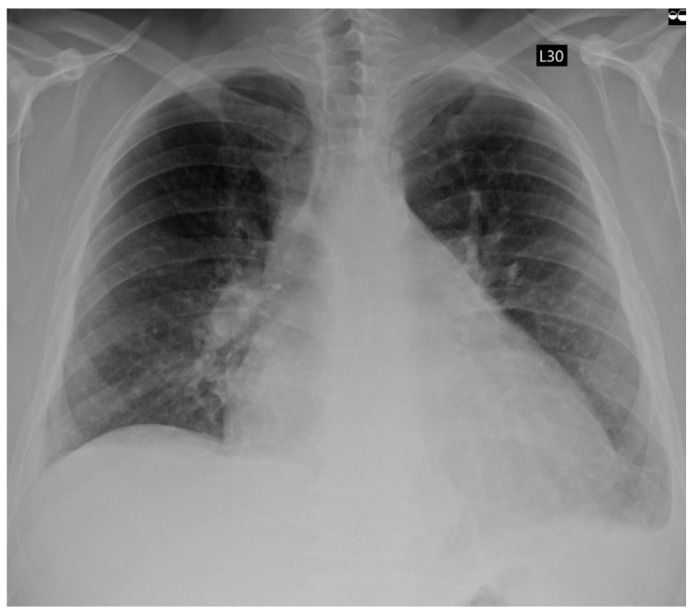
Chest X-Ray showing fluid congestion in admission.

A 42-year-old male patient, previously healthy, physically active, and professionally employed, was referred to the Emergency Department due to progressive exercise intolerance persisting for several weeks. Several days prior, during a visit to a primary care physician, the patient was diagnosed for the first time with AF. Rate control therapy (bisoprolol 5 mg once a day) and oral anticoagulation (apixaban 5 mg twice a day) were initiated. However, despite the implemented treatment, the patient reported no symptomatic improvement. Electrocardiography revealed AF with a rapid ventricular response of approximately 160 beats per minute. Transthoracic echocardiography (TTE) demonstrated globally reduced left ventricular systolic function with a left ventricular ejection fraction (LVEF) of 25%, left ventricular dilatation (LVESD 55 mm, LVEDD 61 mm), left (LA 54 mm, LAA 36 cm^2^; LAVI 58.13 mL/m^2^) and right (RAA 22.1 cm^2^) atrial enlargement, and moderate functional mitral regurgitation. Physical examination revealed signs of congestion in both the systemic and pulmonary circulations. Laboratory investigations showed elevated levels of natriuretic peptides, as well as markers of hepatic and renal dysfunction. Chest radiography demonstrated features of pulmonary congestion and a small amount of pleural effusion in both pleural cavities (Figure 1).

The patient was admitted to the hospital, where guideline-directed medical therapy for heart failure was initiated (bisoprolol 5 mg once a day, eplerenone 50 mg once a day, empagliflozin 10 mg once a day and sacubitril/valsartan 49/51 mg twice a day), also including intensive diuretic treatment, resulting in a reduction in body weight by approximately 13 kg. An attempt of ECV performed during hospitalization was unsuccessful. A decision was made to schedule an elective repeat ECV after pre-treatment with amiodarone, planned approximately 4 weeks later. Unfortunately, this attempt was also ineffective. As part of the diagnostic work-up to determine the underlying cause of heart failure, coronary computed tomography angiography was performed, which excluded the presence of significant hemodynamically relevant coronary artery stenoses. Based on the overall clinical context, TIC (tachycardia-induced cardiomyopathy) was considered the most likely etiology. Following the implemented treatment, the patient experienced resolution of overt acute heart failure symptoms and reported clinical improvement, although he continued to limit his physical activity. In view of the overall clinical picture, an elective AF ablation procedure was scheduled. Approximately two months later, the patient was re-admitted to the department for a PVI procedure using radiofrequency (RF) ablation guided by a three-dimensional electroanatomic mapping system (Ensite X, Abbott). Ablation-specific procedural data are listed in Table 1.

**Figure 2:**
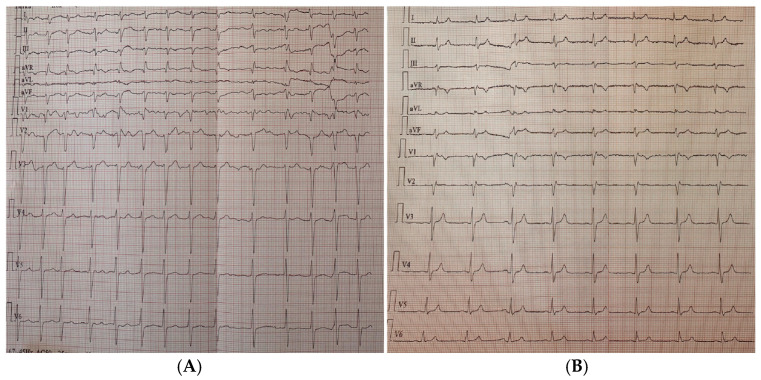
ECG tracings before and after ablation: (**A**) atrial fibrillation recorded during hospital admission for the ablation procedure; (**B**) sinus rhythm observed at follow-up.

At admission, the ECG showed persistent AF with adequate ventricular rate control at approximately 85 bpm (Figure 2).

**Figure 3:**
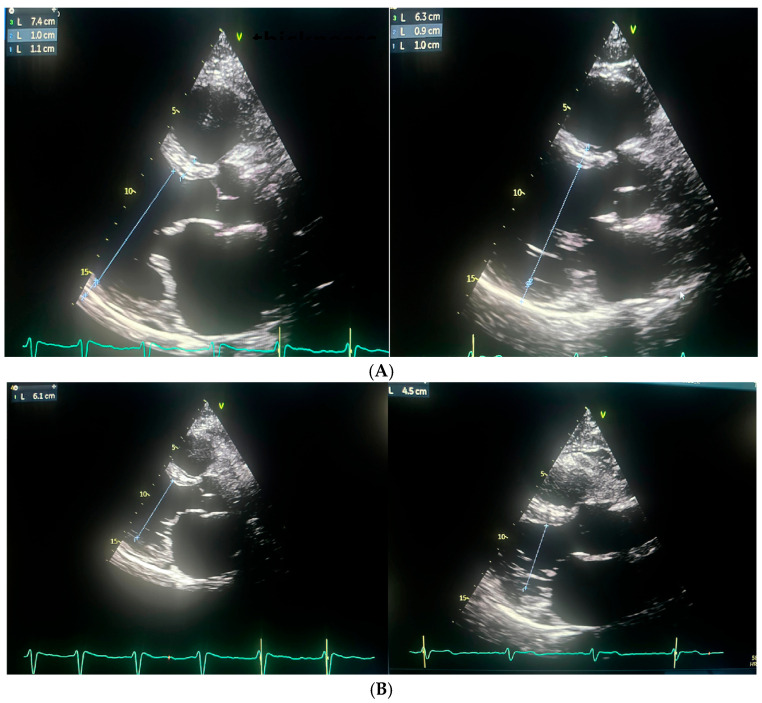

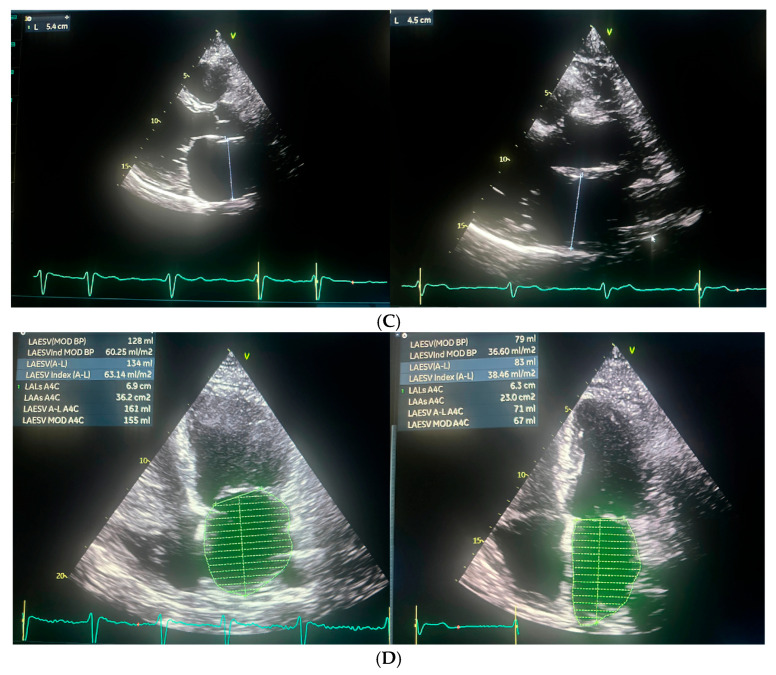
The figure illustrates the changes in cardiac chamber dimensions as assessed by echocardiography, comparing measurements obtained before and after the ablation procedure. Echocardiographic parameters prior to ablation are displayed on the left, while post-ablation values are shown on the right. (**A**) Left Ventricle End Diastolic Volume (LVEDD) and myocardium; (**B**) Left Ventricle End Systolic Volume (LVESD); (**C**) Left atrium postero-lateral diameter; (**D**) Left atrium volume index (LAVI).

**Figure 4:**
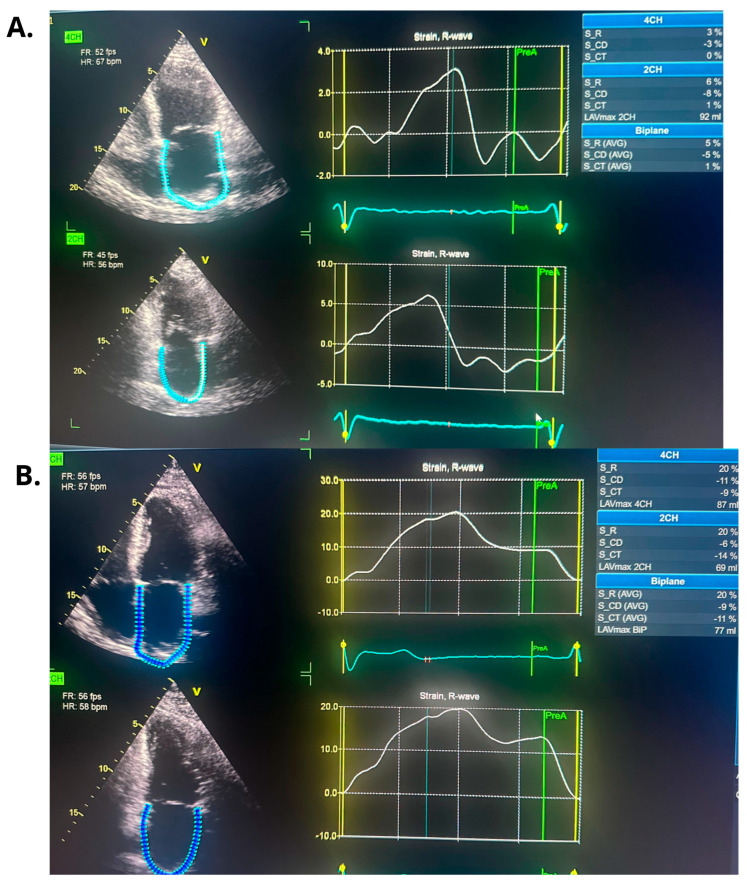
The figure illustrates changes in left atrial strain parameters observed before and following catheter ablation. (**A**). Left atrial strain parameters before ablation (during ongoing atrial fibrillation). (**B**). Left atrial strain parameters after ablation in follow-up time (during sinus rhythm).

Repeated TTE demonstrated a slight improvement in left ventricular systolic function, with an LVEF of 25%. (Figure 3 and Figure 4).

**Figure 5:**
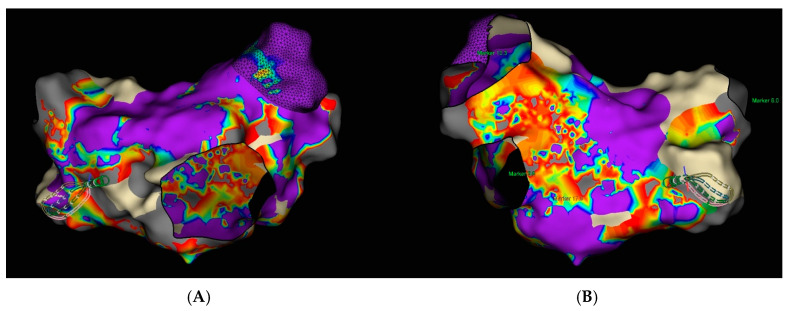
Electroanatomic map of the left atrium during atrial fibrillation created using the Ensite X system (Advisor HD Grid mapping): (**A**) Posterior–Anterior view; (**B**) Anterior–Posterior view.

Transesophageal echocardiography (TEE) was performed to exclude the presence of intracardiac thrombi in the left atrium and left atrial appendage. Under local anesthesia, a diagnostic electrode was introduced via the right femoral vein into the coronary sinus (CS) and to the His bundle region in the right ventricle. A single transseptal puncture was performed, and a diagnostic Advisor HD Grid catheter was advanced into the left atrium through a steerable Agilis sheath. An electroanatomic map of the left atrium was created during ongoing AF (Figure 5).

**Figure 6:**
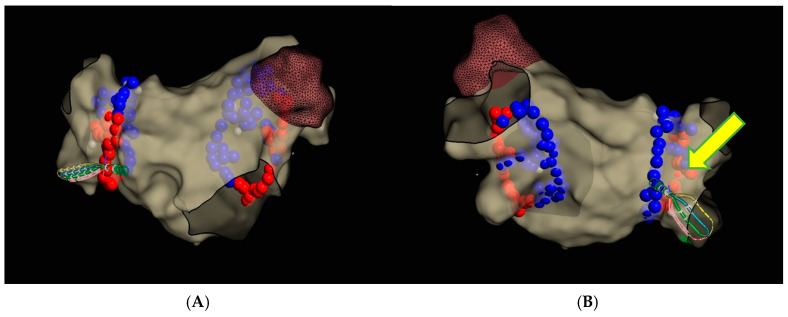
Pulmonary vein isolation. Dots indicate RF ablation sites using the TactiFlex ablation catheter (red dot—20 s, 45 watts; blue dot—15 s, 50 watts). The arrow marks the site where atrial fibrillation terminated and sinus rhythm was restored. (**A**) Anterior–Posterior view; (**B**) Posterior–Anterior view.

Subsequently, circumferential PVI was performed using TactiFlex Ablation Catheter SE (Figure 6) (50 W for 10–15 s on the posterior wall and 45 W for 15–20 s in the other regions, with contact force maintained within the recommended range, 15–20 g).

**Figure 7:**
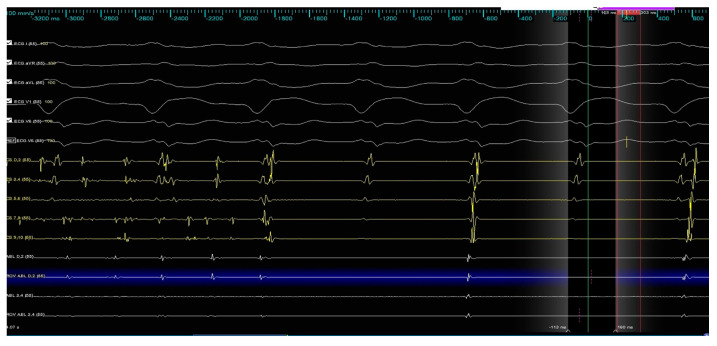
Termination of atrial fibrillation during RF application.

During the ablation of the infero-posterior aspect of the right inferior pulmonary vein, spontaneous restoration of sinus rhythm occurred and was maintained until the end of the procedure (Figure 7).

**Figure 8:**
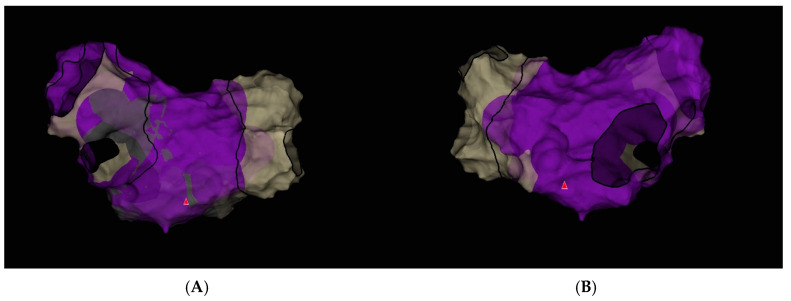
Electroanatomic map of the left atrium during sinus rhythm created using the Ensite X system (Advisor HD Grid mapping) after pulmonary vein isolation. (**A**) Posterior–Anterior view; (**B**) Anterior–Posterior view.

In the final voltage map, no areas of low-voltage, high-amplitude potentials suggestive of additional arrhythmogenic substrate were identified (Figure 8).

Following the ablation procedure, chronic amiodarone therapy was discontinued. The patient was subsequently scheduled for follow-up visits approximately 2 months and 14 months after the intervention. Since the ablation, the patient remained in very good general condition, with no documented recurrences of arrhythmia, either symptomatically or on 24 h Holter ECG monitoring. There were no hospitalizations for arrhythmia-related events during this period. At the 14-month follow-up, TTE was performed and complete normalization of left ventricular systolic function was observed, with an LVEF of 55% (Figure 3 and Figure 4). Additionally, a reduction in the dimensions of both atria, the right and left ventricles, was noted. The previously elevated natriuretic peptide levels had also regressed significantly. Importantly, the patient’s functional status and quality of life improved markedly, as assessed by the ASTA questionnaire, with a score reduction from 24 points before the procedure to 0 points after ablation. General and echocardiographic parameters before and after ablation are presented in Table 2, Table 3 and Table 4 respectively.

## 4. Discussion

Persistent AF resistant to both antiarrhythmic therapy and ECV may be associated with a more advanced arrhythmogenic substrate, and is often associated with poor response to ablation. However, in selected cases, PVI alone—particularly when a single, well-placed radiofrequency application results in immediate AF termination—may suggest a predominantly PV-dependent mechanism (potentially driven by localized micro-reentry in ostial region), even in seemingly unfavorable clinical scenarios [7]. In a computational study, Rappel et al. employed in silico modeling to investigate the mechanisms underlying AF termination by localized ablation. Their simulations demonstrated that targeted ablation could destabilize or eliminate rotors by creating conduction block or forcing wavefront collisions, leading to spontaneous AF termination. These findings support the concept that discrete driver regions can sustain AF, and their strategic elimination may be sufficient for arrhythmia cessation, even without extensive lesion sets [7]. However, Elayi et al. demonstrated that termination of AF during the procedure—defined as conversion to sinus rhythm or another atrial arrhythmia—does not significantly correlate with improved long-term maintenance of sinus rhythm after one or more ablation procedures, compared to patients in whom AF persisted at the end of the ablation. The study emphasizes that the mode of AF termination—whether directly to sinus rhythm or via an intermediate atrial arrhythmia—may predict the type of arrhythmia recurrence, but does not influence overall long-term procedural efficacy [8]. Similarly, a meta-analysis conducted by Wang et al. demonstrated that in patients with long-standing persistent AF, pursuing AF termination during ablation was associated with comparable long-term clinical outcomes when compared to strategies based on achieving a predefined technical endpoint (defined as circumferential pulmonary vein antrum isolation and/or ablation of complex fractionated atrial electrogram, and/or linear ablation between two anatomical structures like the mitral isthmus, left atrial roof) [9]. These findings were also reflected in the 2017 and sustained in the 2024 HRS/EHRA consensus statement, which concluded that there is no conclusive evidence that acute termination of AF during ablation is associated with improved outcomes, including longer arrhythmia-free survival. However, it was suggested that in patients with persistent or long-standing persistent AF—as opposed to paroxysmal AF—such termination may reflect effective modification of the arrhythmogenic substrate [2,10]. Furthermore, the latest 2023 guidelines from the Society of Thoracic Surgeons on the surgical management of AF do not recommend acute AF termination as a procedural endpoint, instead emphasizing hard endpoints such as long-term freedom from arrhythmia [11]. A similar case has been described by Herveg et al., involving acute termination of AF during PVI in a patient with persistent AF resistant to multiple cardioversions and antiarrhythmic therapy, including amiodarone. A single radiofrequency application within the left superior pulmonary vein resulted in immediate AF termination and restoration of sinus rhythm [12]. In contrast to our case, long-term follow-up assessing the effectiveness of acute AF termination was lacking.

There is a growing emphasis on optimizing patient selection for AF ablation, aiming to identify individuals most likely to benefit from the procedure. The i-STRATIFICATION study [13] demonstrated that tailoring ablation strategies based on individual anatomical and electrophysiological characteristics—such as atrial size and presence of low-voltage areas—significantly improves treatment outcomes. Similarly, the TAILORED-AF trial [14] showed that in patients with persistent AF, ablating regions of spatiotemporal dispersion in a structurally normal heart, in addition to PVI, resulted in superior rhythm control compared to PVI alone, supporting the need for a mechanistic, substrate-guided approach. Complementing these findings, the DR-FLASH (Table 4) score has emerged as a practical clinical tool to predict the likelihood of a non-remodeled left atrium in patients with persistent AF. Low DR-FLASH scores (<3 points) are associated with the absence of significant atrial fibrosis or low-voltage areas, helping identify patients in whom PVI alone may be sufficient [15].

In our patient, catheter ablation resulted in a marked improvement of left atrial mechanical function, as assessed by LA strain. While such functional recovery may not be achievable in every patient with persistent atrial fibrillation and impaired left atrial strain [16], it could potentially have meaningful implications for long-term outcomes, including thromboembolic events and stroke, as suggested by available literature [17,18].

These insights collectively support the idea that even patients with longstanding, cardioversion-resistant AF could respond favorably to ablation if appropriately selected using such stratification tools. Analysis of the electrograms recorded under the ablation catheter did not reveal fragmented signals with high-frequency activity that would typically suggest a focal trigger mechanism; however, the exact mechanism of AF termination, whether focal trigger or local micro-reentry, remains speculative. Our patient was a young individual with no comorbidities and a low DR-FLASH score (2 points), which supported the absence of advanced substrate within the left atrium. This suggests that, despite an unfavorable clinical presentation, the likelihood of a successful ablation outcome remains high and is commonly achievable with PVI alone.

## 5. Conclusions

This case illustrates that even in patients with persistent/therapy-resistant AF and a truly unfavorable clinical setting (significant atrial enlargement, left ventricular dilatation with low LVEF, moderate mitral regurgitation), discrete localized regions may sustain the arrhythmia, and their targeted elimination during ablation can lead not only to acute arrythmia termination, but also to a durable maintenance of sinus rhythm during long-term follow-up.

## Figures and Tables

**Table 1:** Ablation-specific procedural data.

Ablation Parameters	Value
Skin to skin time (s)	168
Left atrium dwell time (s)	141
Ablation time (s)	83
Mean ACT time (s)	310
Fluoroscopy time (s)	682
Dose area product (mG)	215

**Table 2:** General patient parameters before and after ablation.

General Parameters	First Admission	Ablation	Follow-Up
Day of analysis	0	60	529
Weight (kg)	123	98	100
BMI (kg/m^2^)	40.16	32	32.65
NYHA class	IV	III	I
EHRA	IV	III	I
NTproBNP (pg/pL)	3948	4164	213
Hemoglobin (g/dL)	14.6	17.3	14.8
Creatinine (mg/dL)	1.06	1.2	0.99
Potassium (mmol/L)	4.5	4.2	4.4
CRP (mg/L)	12.2	4.9	0.8

**Table 3:** Echocardiographic parameters during acute decompensation of heart failure, during elective hospitalization for ablation, and during scheduled follow-up (LVEF—left ventricular ejection fraction; LVEDD—left ventricular end diastolic dimension; LVESD—left ventricular end systolic dimension; LA—left atrium; LAA—left atrium area; LAVI—left atrium volume index; RAA—right atrium area; TAPSE—tricuspid annular plane systolic excursion; PALS—peak atrial longitudinal strain; RVSP—right ventricular systolic pressure).

Echocardiographic Parameters	First Admission	Ablation	Follow-Up
LVEF (%)	25	27	54
LVEDD (mm)	61	74	63
LVESD (mm)	55	61	45
LA (mm)	48	54	45
LAA (cm^2^)	-	36.2	23
LAVI (ml/m^2^)	-	60.25	36.6
RAA (cm^2^)	-	22.8	21.5
TAPSE (mm)	-	18	23
PALS (%)	-	5	20
RVSP (mmHg)	37	40	-

**Table 4:** DR-FLASH Score Components (total score range: 0–7).

Component	Points
Diabetes mellitus	1
Renal dysfunction	1
Persistent atrial fibrillation	1
Left atrial diameter > 45 mm	1
Age > 65 years	1
Female sex	1

## Data Availability

The data supporting the report are available from Jonasz Kozielski (jonaszkozielski@gmail.com) on reasonable request.
